# Diet quality trends among adults with diabetes by socioeconomic status in the U.S.: 1999–2014

**DOI:** 10.1186/s12902-019-0382-3

**Published:** 2019-05-31

**Authors:** Colin J. Orr, Thomas C. Keyserling, Alice S. Ammerman, Seth A. Berkowitz

**Affiliations:** 1 Department of Pediatrics, School of Medicine, University of North Carolina, Chapel Hill, NC USA; 2 Cecil G. Sheps Center for Health Services Research, University of North Carolina, Chapel Hill, NC USA; 3 Division of General Medicine and Clinical Epidemiology, Department of Medicine, School of Medicine, University of North Carolina, Chapel Hill, NC USA; 4 Center for Health Promotion and Disease Prevention, University of North Carolina, Chapel Hill, NC USA; 5 Department of Nutrition, Gillings School of Global Public Health and School of Medicine, Chapel Hill, NC USA

**Keywords:** Diabetes mellitus, Healthcare disparities, Diet, Nutrition survey

## Abstract

**Background:**

The diet quality of adults living in the United States has improved overtime. We aim to determine whether diet quality among adults with diabetes mellitus has changed over time, and to examine trends in socioeconomic disparities in diet quality.

**Methods:**

Repeated cross-sectional analysis of eight National Health and Nutrition Examination Survey (NHANES) cycles (1999–2000 through 2013–2014). We included 5882 adult participants (age 20 or older) with diabetes mellitus (type 1 or 2) who completed 24-h dietary recalls. Diet quality was measured by the Healthy Eating Index 2010 (HEI) score (range 0–100, higher scores indicate better diet quality). We tested whether there were differences in diet quality across education, income, and food security categories, and whether any differences changed over time, using weighted linear regression models accounting for the complex survey design and adjusted for age, gender, and race/ethnicity.

**Results:**

Twenty nine percent of US adults with diabetes had less than a high school diploma, 17% had income < 100% of federal poverty level, and 15% reported food insecurity. Average adjusted HEI score increased from 49.4 to 52.4 over the study period (p for trend = 0.003). We observed differences in HEI between high and low education (4.1, 95% CI 3.0–5.3), high and low income (3.7, 95%CI 2.4–5.0) and food secure relative to food insecure (2.1, 95% CI 0.8–3.3). These differences did not improve over time for education (*p* = 0.56), income (*p* = 0.65) or food security (*p* = 0.39) categories.

**Conclusions:**

Diet quality for adults with diabetes in the U.S. has improved overall; however, substantial disparities exist and have not improved. A concerted effort to improve diet quality in vulnerable groups may be needed.

**Electronic supplementary material:**

The online version of this article (10.1186/s12902-019-0382-3) contains supplementary material, which is available to authorized users.

## Background

Diet quality is important for individuals with diabetes mellitus. The American Diabetes Association recommends “healthful eating patterns, emphasizing a variety of nutrient-dense foods” to help “attain individual glycemic, blood pressure, and lipid goals” and to “delay or prevent the complication of diabetes.” [1] With regard to complications of diabetes, and in particular cardiovascular disease (CVD) outcomes, which are more common in those with diabetes [2, 3], emerging data underscore the importance of dietary pattern for those with diabetes. In observational studies focusing on participants with diabetes at baseline, a higher quality diet was associated with decreased rates of CVD events and CVD mortality [4] and total mortality [5].

 Among all US adults, diet quality has increased over the last decade [6]; however, disparities in diet quality between individuals with high and low socioeconomic status have increased [6]. Differences in diet quality may be an important explanation for socioeconomic disparities in diabetes-related health outcomes, including glycemic control, CVD events, and mortality [7, 8]. Notably, individuals with diabetes and lower socioeconomic status have as much as a two-fold greater risk of mortality relative to those with greater socioeconomic status [8, 9].

Despite the importance of diet quality on diabetes-related health outcomes and disparities in outcomes, the extent of socioeconomic differences in diet quality among individuals with diabetes is not well studied. Further, whether the differences in diet quality have changed over time is unknown. In this study, using repeated cross-sections of nationally-representative data, we investigate trends in diet quality among adults with diabetes. We hypothesize that individuals with indicators of lower socioeconomic status will have lower diet quality, and that this difference will not have improved over time.

## Methods

### Study design and sample

We used data from 8 cycles of the National Health and Nutrition Examination Survey (NHANES) (covering the years 1999 through 2014) for this analysis. These cycles go up to the most recent data available that include all variables of interest. NHANES is a nationally-representative sample of community dwelling Americans. More extensive description of the design and methods of NHANES is available on the NHANES website [10]. We included adults (20 years of age or older) [11] who had diabetes mellitus (type 1 or type 2). Similar to prior studies, participants were classified as having diabetes by self-report, random glucose ≥200 mg/dL, fasting glucose ≥126 mg/dL, HbA1c ≥6.5%, or use of a glucose lowering medication other than metformin such as a sulfonylurea, insulin, or incretin mimetic [12]. Metformin use as single agent was not considered diagnostic owing to its use in adults without diabetes. The institutional review board (IRB) at the University of North Carolina at Chapel Hill considered our use of de-identified data for this study as exempted from IRB review.

### Outcome measure: diet quality

The outcome for this study was diet quality as measured by the Healthy Eating Index (HEI) 2010. The HEI 2010 has been described in detail previously [13]. Briefly, the HEI measures diet quality according to the Dietary Guidelines for Americans, 2010 [13]. The HEI 2010 is comprised of 12 component scales (range 0–5, 0–10, or 0–20), which are combined to produce total HEI 2010 score (range 0–100). For all scales, higher numbers indicates better diet quality [13]. This means that higher consumption of desirable foods, such as fruits and vegetables, and lower consumption of less desirable diet components, such as sodium or added sugars, lead to higher scores. A list of the component scales and their range is presented in Additional file 1: Table S1.

The HEI 2010 has been validated in previous research [14] and shown to be associated with diabetes prevalence [15], glycemic control [7], and with risk for a variety of other common chronic diseases [15, 16]. Nutritional data used to calculate HEI 2010 were obtained using the NHANES dietary recall assessment. In accordance with guidelines for the assessment of population diet quality [17], we calculated, using computer code provided by the National Cancer Institute, HEI scores using a single 24-h dietary recall. The 24-h dietary recall information was collected by a trained interviewer in English or Spanish [18].

### Socioeconomic status indicators

We used two socioeconomic status indictors in our analysis: education and income. Education was categorized as less than high school diploma, high school diploma or equivalent, and more than a high school diploma. To account for both inflation and household size, income was expressed as percentage of the ratio of household income to the federal poverty level for the household size in the year the data were collected (poverty to income ratio or PIR). This divides the participant’s household income by the applicable poverty threshold for the year of data collection and for a household of the participant’s size. For example, the federal poverty level for a family of four in 2019 is $25,750, and if the participant’s income was $32,125 and they had a household size of 4, their PIR value would be 1.25, or 125% of the federal poverty level. The PIR was categorized as less than 100%, 100–200%, and greater than 200% of the federal poverty level. In addition to these socioeconomic status indicators, we examined the related issue of food insecurity, defined as limited or inconsistent access to nutritious food owing to cost [19]. While not a socioeconomic status indicator itself, food insecurity has been associated with a number of poor diabetes outcomes, which may be related to changes in diet quality induced by food insecurity [7, 12]. Food insecurity was assessed using the 10 adult referenced items of the USDA Household Food Security Survey Module [20]. Three or more affirmative responses indicated food insecurity.

### Covariates

For descriptive purposes, we considered several other covariates. Age, race/ethnicity (categorized as Mexican-American, other Hispanic, Non-Hispanic White, Non-Hispanic Black and Other-Race), and gender (categorized as male or female) were obtained by self-report. We considered glucose lowering medications (reported by participants and confirmed by inspection of pill bottles by interviewers) in four categories (metformin alone, sulfonylurea use alone, use of more than one glucose lowering medication but no insulin, and use of insulin with or without any other glucose lowering medications). We also identified use of statins and use of angiotensin converting enzyme (ACE) inhibitors. Hemoglobin A1c, body mass index (BMI), systolic and diastolic blood pressure, total cholesterol, high-density lipoprotein cholesterol, low-density lipoprotein cholesterol, and triglycerides were obtained via measurement following standard NHANES protocols [10].

### Statistical analysis

The main goal of our analysis was to determine whether diet quality differed by socioeconomic/food security indicators, and whether any differences varied over time. As recommended in the NHANES guidelines for analysis of trends, we conducted a record-level analysis using linear regression [21]. All analyses used survey weights and clustering information to account for complex survey design. Since the outcome of interest was diet quality, we used the dietary weights in order to generate nationally representative estimates. Descriptive statistics were used to describe the overall study population as well as the study population for each NHANES cycle, treated as ordered categorical variable (1999–2000, 2001–2002, 2003–2004, 2005–2006, 2007–2008, 2009–2010, 2011–2012, and 2013–2014). To examine differences in diet quality between groups compared to a referent group, we fit linear regression models with socioeconomic exposure variable and NHANES cycle as independent variables, and total HEI 2010 score as the dependent variable. We then used least square means from these models to estimate adjusted mean HEI 2010 scores. Because individuals may modify their diet in response to a diagnosis of diabetes, we also conducted subset analyses separately examining those who self-report a diagnosis of diabetes, and those who did not (and thus had diabetes on the basis of laboratory measurements only). To facilitate comparisons over time, linear regression models were adjusted for race/ethnicity, gender and age. To test whether differences changed over time, we fit additional linear regression models with an exposure-by-NHANES cycle interaction term (in addition to the main terms), also adjusted for race/ethnicity, gender and age. Because our goal was to understand the diet quality of groups defined by socioeconomic status/food insecurity indictors, rather than to determine whether these indicators were ‘risk factors’ for poor diet quality, we did not adjust for additional covariates.

Finally, to guide future study, we sought to describe the diet quality, stratified by socioeconomic status and food insecurity indicators, of the most recent NHANES cycle. We view these analyses as exploratory, and did not conduct statistical testing of these patterns for this reason, along with concerns about multiple testing. Because missingness for the variables of interest was less than 10%, missing data were not imputed. Statistical analyses were conducted using Stata IC version 14.1 (College Station, TX).

## Results

A total of 5882 participants were included in the analysis (Table 1, participants by diabetes diagnostic criteria Additional file 1: Table S2). Women represented 49.2% (weighted) of those included, and the average age of the population the sample represents was 59.2 (SE 0.3) years. Non-Hispanic whites represented 61.8% of the population, 15.9% were non-Hispanic black, 8.9% were Mexican-American, 5.9% were “other” Hispanic and 7.5% were other race/ethnicity. Twenty nine percent of the represented population had less than a high school diploma, 25.7% had a high school diploma and 45.2% had more than a high school diploma. Poverty income ratio (PIR) of < 100% was reported by 17.4, 27.4% had a PIR of 100–200 and 55.3% had a PIR > 200%. Almost 15% reported food insecurity. Overall, mean BMI was 32.8 (32.4–33.1) and increased from 31.9 (31.0–32.8) in 1999–2000 to 33.5 (32.6–34.4) in 2013–2014 (Additional file 1: Table S3). Mean glycosylated hemoglobin decreased from 7.78 (7.42–8.15) in 1999–2000 to 7.18 (7.00–7.35) in 2013–2014. Additional laboratory and medication use information is presented in Additional file 1: Table S3.

**Table 1:** Demographics Representing US Adults with Diabetes

	Overall	1999–2000	2001–2002	2003–2004	2005–2006	2007–2008	2009–2010	2011–2012	2013–2014
Unweighted N	5882	554	597	614	594	943	938	806	836
Weighted N	23,290,811	2,061,780	2,317,982	2,711,120	2,633,596	3,163,996	3,160,395	3,390,174	3,851,764
	Mean/ Percent	Mean/ Percent	Mean/ Percent	Mean/ Percent	Mean/ Percent	Mean/ Percent	Mean/ Percent	Mean/ Percent	Mean/ Percent
	(95%CI)	(95%CI)	(95%CI)	(95%CI)	(95%CI)	(95%CI)	(95%CI)	(95%CI)	(95%CI)
Age (Years)	59.2	58.6	58.2	59.4	59.6	59.3	60.0	59.4	58.8
	(58.6–59.8)	(56.4–60.8)	(56.0–60.4)	(57.3–61.5)	(57.0–62.2)	(58.2–60.4)	(58.5–61.4)	(58.3–60.5)	(57.8–59.9)
Female	49.2	49.3	48.0	47.3	54.6	49.6	47.1	48.6	49.3
	(47.5–50.9)	(43.1–55.4)	(44.2–51.9)	(43.0–51.6)	(48.1–61.0)	(44.6–54.6)	(42.8–51.4)	(43.8–53.4)	(45.8–52.9)
Race/ Ethnicity									
Mexican American	8.9	6.4	7.2	7.6	8.9	9.0	11.6	8.7	10.1
	(7.0–10.8)	(2.3–10.6)	(4.4–10.0)	(1.3–14.0)	(5.7–12.0)	(4.9–13.2)	(4.6–18.5)	(2.9–14.6)	(5.1–15.1)
Other Hispanic	5.9	9.3	6.7	3.3	4.8	5.1	5.6	8.5	4.8
	(4.4–7.4)	(0.0–19.4)	(0.4–13.1)	(0.7–5.8)	(2.7–6.9)	(2.4–7.9)	(2.4–8.8)	(4.5–12.4)	(2.9–6.8)
NH White	61.8	60.7	63.5	68.6	63.1	63.3	60.3	55.1	61.8
	(58.6–65.1)	(49.8–71.5)	(55.1–71.8)	(59.4–77.9)	(54.6–71.7)	(52.2–74.4)	(52.0–68.6)	(45.9–64.3)	(54.8–68.9)
NH Black	15.9	16.5	14.9	13.4	18.6	16.7	16.0	17.2	14.1
	(13.9–17.9)	(8.1–24.9)	(9.6–20.3)	(9.1–17.7)	(13.0–24.3)	(11.0–22.3)	(12.3–19.7)	(10.1–24.4)	(9.3–19.0)
Other Race	7.5	7.1	7.7	7.0	4.6	5.9	6.5	10.4	9.1
	(6.1–8.8)	(0.3–13.9)	(1.9–13.5)	(4.3–9.8)	(1.2–8.0)	(2.3–9.5)	(4.5–8.6)	(6.3–14.7)	(6.3–11.9)
Education									
<HS	29.1	41.9	32.3	30.0	26.2	30.1	29.8	27.8	21.2
	(27.1–31.0)	(33.1–50.6)	(26.7–37.9)	(24.7–35.4)	(20.9–31.5)	(25.7–34.6)	(26.5–33.1)	(22.3–33.3)	(16.0–26.3)
HS	25.7	30.1	22.6	23.0	30.6	27.9	21.6	25.9	25.2
	(23.8–27.7)	(20.9–39.4)	(18.7–26.5)	(19.3–26.7)	(25.1–36.2)	(21.8–33.9)	(17.3–26.0)	(19.7–32.2)	(21.5–28.9)
>HS	45.2	28.0	45.1	47.0	43.2	42.0	48.6	46.2	53.7
	(42.9–47.5)	(22.8–33.2)	(39.3–50.8)	(41.1–52.8)	(34.1–52.3)	(35.7–48.3)	(44.5–52.6)	(39.0–53.5)	(48.1–59.2)
Poverty to Income Ratio									
<100%	17.4	24.2	17.4	15.3	13.9	15.6	14.5	22.5	17.0
	(15.7–19.1)	(15.6–32.7)	(13.2–21.6)	(10.8–19.7)	(9.3–18.5)	(12.0–19.3)	(10.3–18.6)	(17.9–27.1)	(12.6–21.4)
100–200%	27.4	28.8	26.9	28.3	28.7	29.0	25.5	28.6	24.5
	(25.5–29.2)	(25.3–32.4)	(21.5–32.4)	(21.9–34.6)	(23.9–33.5)	(25.4–32.5)	(21.5–29.6)	(23.0–34.2)	(18.5–30.5)
>200%	55.3	47.0	55.7	56.5	57.4	55.4	60.0	48.9	58.5
	(52.9–57.6)	(40.4–53.7)	(48.5–62.8)	(48.3–64.6)	(49.6–65.1)	(50.3–60.4)	(54.1–65.9)	(41.3–56.5)	(53.6–63.3)
Food Insecurity	14.6	10.8	12.7	11.8	10.3	13.6	16.1	19.4	17.7
	(13.2–15.9)	(6.9–14.8)	(10.4–14.9)	(8.9–14.8)	(7.2–13.3)	(10.5–16.7)	(11.9–20.3)	(15.2–23.5)	(13.6–21.8)

Abbreviations: *NH* Non-Hispanic, *HS* High School; Poverty to Income Ratio represents ratio of participant’s household income to federal poverty threshold in year of data collection, accounting for household size

**Fig. 1:**
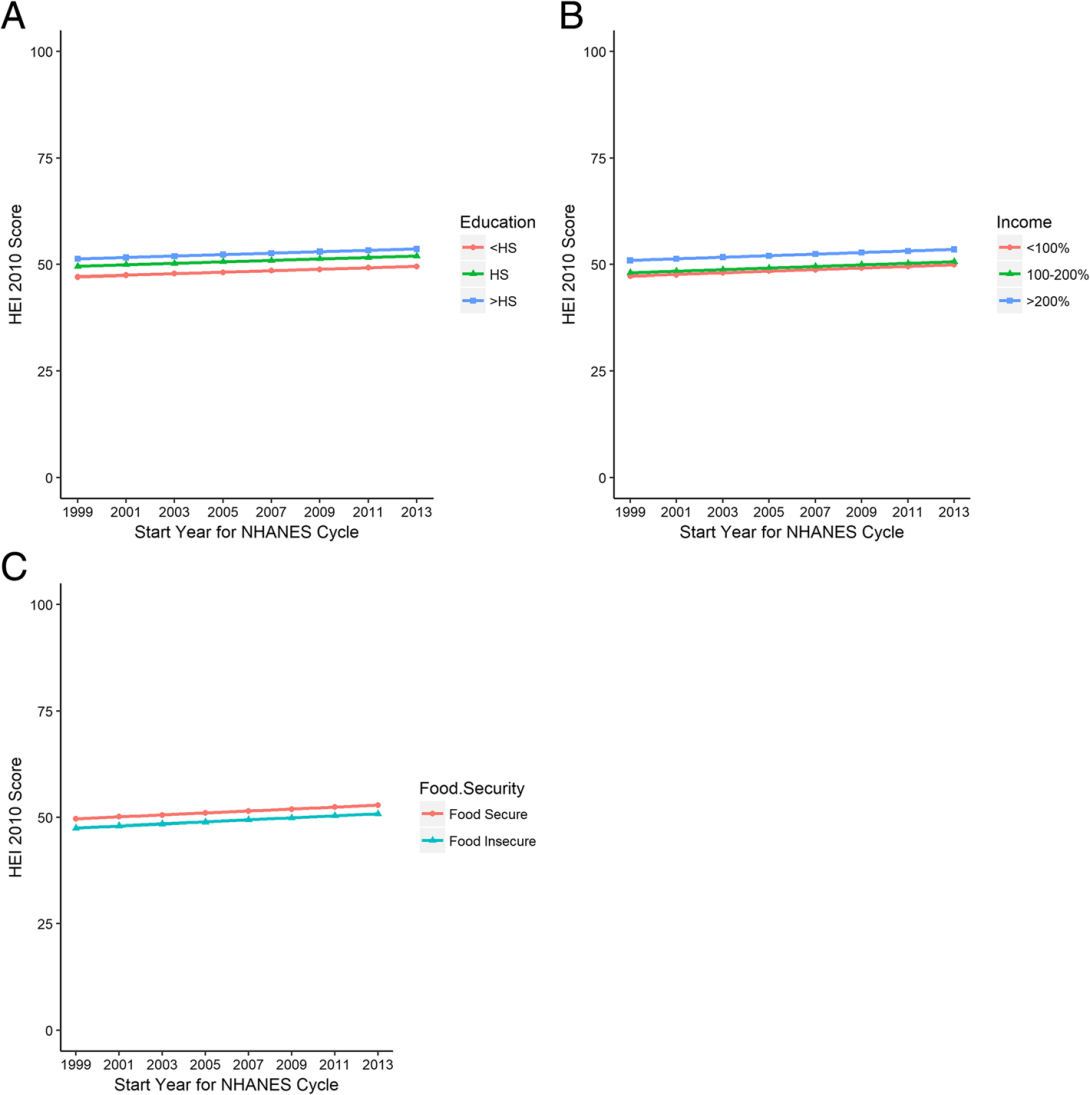
**a**-**c** shows the results of mean Healthy Eating Index 2010 score by NHANES cycle stratified by level of education (**a**), Income expressed as the ratio of household income to the federal poverty threshold and converted to a percentage, (**b**), and Food Security Status (**c**). Results are from weighted linear regression models, adjusted for age, gender, and race/ethnicity. HS = high school

The weighted and adjusted mean HEI 2010 score over the entire study period was 51.3 (95% CI 50.7 to 51.9) (Table 2, unadjusted results in Additional file 1: Table S4). The mean HEI score for individuals with diabetes increased from 49.4 (95% CI 48.3–50.5) in the 1999–2000 cycle to 52.4 (95%CI 51.1–53.6) in 2013–2014 cycle (*p*-value for trend = 0.003). US adults with diabetes with more than a high school diploma had a mean HEI score 4.14 (95% CI 2.98 to 5.29, p-value < 0.001) points higher than individuals with less than a high school diploma (Table 3). This difference in diet quality by education did not change over time (p for differential change over time as tested by an interaction term = 0.56) (Fig. 1a).

**Table 2:** Adjusted Mean Healthy Eating Index Score by Education, Income and Food Security Status

Variable (N)	Overall	1999–2000	2001–2002	2003–2004	2005–2006	2007–2008	2009–2010	2011–2012	2013–2014
	Mean HEI (95%CI)	Mean HEI (95%CI)	Mean HEI (95%CI)	Mean HEI (95%CI)	Mean HEI (95%CI)	Mean HEI (95%CI)	Mean HEI (95%CI)	Mean HEI (95%CI)	Mean HEI (95%CI)
Overall (5882)	51.3 (50.7–51.9)	49.4 (48.3–50.5)	49.8 (48.9–50.7)	50.2 (49.5–50.9)	50.7 (50.0–51.3)	51.1 (50.4–51.8)	51.5 (50.7–52.3)	52.0 (50.9–53.0)	52.4 (51.1–53.6)
Education (5870)									
<HS%	48.8 (47.8–49.8)	47.1 (45.8–48.5)	47.5 (46.3–48.7)	47.8 (46.7–48.9)	48.1 (47.1–49.2)	48.5 (47.4–49.6)	48.8 (47.7–50.0)	49.2 (47.9–50.5)	49.5 (48.0–51.0)
HS%	51.2 (50.2–52.3)	49.5 (48.2–50.9)	49.9 (48.7–51.1)	50.2 (49.1–51.3)	50.6 (49.5–51.6)	50.9 (49.8–52.0)	51.3 (50.1–52.4)	51.6 (50.3–52.9)	51.9 (50.5–53.4)
>HS%	52.9 (52.1–53.8)	51.3 (50.1–52.5)	51.6 (50.6–52.6)	51.9 (51.0–52.9)	52.3 (51.4–53.1)	52.6 (51.7–53.5)	53.0 (52.0–54.0)	53.3 (52.1–54.5)	53.6 (52.3–55.0)
Poverty to Income Ratio (5357)									
<100%	49.0 (47.7–50.3)	47.3 (45.7–48.9)	47.6 (46.2–49.1)	48.0 (46.7–49.4)	48.4 (47.1–49.7)	48.7 (47.4–50.1)	49.1 (47.7–50.6)	49.5 (47.9–51.1)	49.9 (48.1–51.6)
100–200%	49.7 (48.6–50.8)	48.0 (46.5–49.5)	48.4 (47.1–49.7)	48.7 (47.6–49.9)	49.1 (48.0–50.2)	49.5 (48.4–50.6)	49.9 (48.6–51.1)	50.2 (48.9–51.6)	50.6 (49.1–52.1)
>200%	52.6 (51.9–53.4)	50.9 (49.7–52.2)	51.3 (50.3–52.3)	51.7 (50.8–52.5)	52.0 (51.3–52.8)	52.4 (51.6–53.2)	52.8 (51.9–53.6)	53.1 (52.1–54.2)	53.5 (52.2–54.8)
Adult Food Security 5791)									
Food Insecure	49.5 (48.4–50.6)	47.5 (46.0–49.1)	48.0 (46.6–49.4)	48.4 (47.2–49.7)	48.9 (47.8–50.0)	49.4 (48.3–50.4)	49.8 (48.7–50.9)	50.3 (49.1–51.5)	50.8 (49.4–52.1)
Food Secure	51.6 (50.9–52.3)	49.6 (48.5–50.7)	50.1 (49.2–50.9)	50.5 (49.8–51.3)	51.0 (50.3–51.7)	51.4 (50.7–52.2)	51.9 (51.0–52.8)	52.4 (51.3–53.5)	52.8 (51.5–54.1)

Abbreviations: *HEI* Healthy Eating Index, *HS* High School; Poverty to Income Ratio represents a ratio of participant’s household income to federal poverty threshold in year of data collection, accounting for household size. Results are weighted means estimated from linear regression models with terms for education, income, or food insecurity along with NHANES cycle, and adjusted for gender, age, and race/ethnicity

**Table 3:** Statistical Testing for Differences in HEI-2010 Score by Education, Income, and Food Security

Socioeconomic Indicator	Difference from Reference Group (95% CI)	*p*-value	Change in HEI-2010 per year (95% CI)	Trend *p*-value	Interaction Coefficient (95% CI)	Interaction *p*-value
Education			0.17 (0.04 to 0.31)	0.01		
<HS	Ref	n/a			Ref	n/a
HS	2.42 (1.13 to 3.71)	<.001			−0.06 (−0.32 to 0.20)	0.66
>HS	4.14 (2.98 to 5.29)	<.001			−0.07 (− 0.31 to 0.16)	0.56
Poverty to Income Ratio			0.18 (0.04 to 0.33)	0.01		
< 100%	Ref	n/a			Ref	n/a
100–200%	0.74 (−0.79 to 2.27)	0.34			−0.10 (− 0.45 to 0.26)	0.59
>200%	3.65 (2.35 to 4.95)	<.001			−0.06 (− 0.33 to 0.21)	0.65
Food Security Status			0.23 (0.09 to 0.37)	0.002		
Food Insecure	Ref	n/a			Ref	n/a
Food Secure	2.06 (0.81 to 3.31)	0.002			0.12 (−0.16 to 0.42)	0.39

Abbreviation: *HEI-2010* Healthy Eating Index 2010, *HS* High School; Poverty to Income Ratio represents a ratio of participant’s household income to federal poverty threshold in year of data collection, accounting for household size. Results are from weighted linear regression model adjusted for race/ethnicity, year, age and gender. Models incorporate survey design information for standard errors and use dietary weights for representativeness. *P*-values are from t-statistics of regression coefficients. NHANES cycle was treated as an ordered categorical variable. For interaction testing, an interaction term between socioeconomic exposure and NHANES cycle was added to the ‘main effects’ model described above

Similarly, US adults with a PIR > 200% had a higher mean HEI score than individuals with PIR < 100% (3.65 points higher, 95%CI 2.35 to 4.95, *p* < 0.001). Again, this difference did not change over time (p for interaction = 0.65) (Fig. 1b).

Adults with diabetes who were food secure had higher mean HEI score than those who were food insecure (2.06 points higher, 95% CI 0.81 to 3.31, *p* = 0.002). Diet quality improved over time for both groups (p = 0.002); however, the difference in diet quality between groups did not change over time (p for interaction = 0.39) (Fig. 1c).

When the analyses were stratified by method of diabetes diagnosis, we found that the unadjusted mean HEI score for those who self-reported a diagnosis of diabetes was 51.7 (95%CI 51.0 to 52.4), and the mean HEI score for those who were diagnosed with diabetes on the basis of laboratory values only was 48.0 (95% CI 47.0 to 49.1) (Additional file 1: Table S5 and Additional file 1: Table S6). In both of these subsets, the magnitude of the difference within each socioeconomic exposure was similar between the groups and followed the pattern of lower education, income, or food insecurity having lower diet quality. There was no differential improvement in diet quality over time for education, income, or food insecurity, compared with better off individuals, in either subset.

Examining the diet quality of the most recent NHANES cycle (years 2013–2014), relevant patterns included higher scores for fruit and vegetable consumption in more well-off groups, and lower scores for refined grains and ‘empty calories’ (solid fats and added sugars) in worse-off groups (Additional file 1: Table S7), but we did not conduct statistical testing of these differences. Box and whisker plots showed that the distribution of HEI scores was similar across groups, though tended to be shifted towards lower scores for those in less well-off groups (Additional file 1: Figure S1a-c).

## Discussion

This study found that over a 16 year period (comprising 8 NHANES cycles from 1999 to 2000 to 2013–2014) adults with diabetes have experienced a modest improvement in diet quality as measured by HEI 2010 scores. However, we found persistent disparities between individuals with higher versus lower socioeconomic status and no evidence that these disparities were improving over time. Further, average diet quality scores indicated that dietary patterns could be substantially improved. To put the magnitude of the disparity in perspective, the mean HEI score for individuals with low income observed near the end of the study period was still lower than the mean HEI score for those with higher income at the beginning of the study. This suggests that at the current pace of improvement, those with lower socioeconomic status are approximately 15 years behind the diet quality of their higher socioeconomic status (SES) counterparts.

The findings of this study should be incorporated into the overall body of work that examines diet quality and disparities in diet quality among individuals with diabetes. A substantial literature relates lower diet quality to worse health outcomes both overall [15, 22, 23] and specifically in individuals with diabetes [4, 5]. Further, randomized trials of dietary interventions in individuals with diabetes have shown that healthy dietary patterns can reduce cardiovascular disease risk [24]. However, recent trends in the pattern of socioeconomic disparities in diet quality among individuals with diabetes are not promising.

Diet quality interventions for low SES individuals with diabetes may be needed to help improve the disparities observed. Interventions to improve diet quality should recognize and address economic and social barriers to changing dietary behaviors, and focus on the type of dietary change most likely to benefit those with diabetes [25]. As protein intake is considered adequate for most Americans including those with diabetes [25], efforts could focus instead on improving fat and carbohydrate quality because higher quality fat and carbohydrate intake is strongly supported by past research as important for health outcomes [4, 5, 26, 27].

Importantly, it is possible to achieve higher dietary quality without significantly increasing cost, as recently demonstrated in a community-based study largely enrolling low-income participants [28]. Early development of healthy eating habits can be difficult in a stressed social environment [29, 30], but there is evidence that cooking and sharing meals at home is associated with better diet quality [31]. These observations suggests a potential role for educational interventions as a way to address SES disparities in diet quality while recognizing socioeconomic factors may not be easily changed—and that individuals with lower SES should not be blamed for the factors that promote unhealthy diets.

In terms of specific foods, common vegetable oils (canola, soybean, corn, and peanut) are relatively high in polyunsaturated fats, and are contained in inexpensive, familiar, and easily accessible foods (e.g. full fat salad dressing, mayonnaise). Furthermore, nuts have high quality fats and inexpensive peanuts and peanut butter have benefits similar to tree nuts [32]. Consuming these healthful, yet inexpensive foods may reduce the barrier of cost for low-income families. Other cost-minded changes include consuming whole grain products (bread, rice, etc.) instead of their refined counterparts, eating more beans, and using frozen instead of fresh fruits and vegetables.

Contextually, the increase in HEI score among participants in this study was similar to improvements in the HEI score among adults living in the United States overall [6]. For US adults, the overall mean HEI in 1999–2000 was 46.6 (45.0–48.2) with an increase to 49.6 (48.9–50.4) in 2009–2010 [6]. The higher HEI score among those in this study compared to estimates of the overall US population may be due to greater dietary counseling provided patients with diabetes relative to the general population. Despite improvement in diet quality over time, BMI increased over the course of this study, a temporal trend observed in other studies among those with diabetes [33, 34] and without diabetes [35]. This is likely attributable to multiple individual and environmental factors [36]. Additionally, we observed a decrease in glycosylated hemoglobin over time, consistent with temporal trends of earlier diagnosis and more intensive medication management for adults with diabetes [37, 38].

This study suggests several directions for future research. Additional analyses could explore the trends in diet quality by other aspects of diabetes such as: type of diabetes, duration of diabetes, medication use, HbA1c, and BMI. These analyses would help advance the care of adults with diabetes and potentially identify groups that need additional support in improving diet quality. Beyond these observational studies, testing dietary interventions for individuals with diabetes and low SES is of the utmost importance. A recent randomized trial found improvements in diet quality, though no improvements in glycemic control over a relatively short time period, for food pantry participants with diabetes [39]. Further work can build on interventions like these to support individuals with diabetes in following a healthy diet, especially when presented with the competing demands that those with lower SES may additionally face, such as lack of money for medications or transportation barriers [40].

The results of this study should be considered in the context of several limitations. NHANES uses a repeated cross-section design, which means we cannot observe changes in specific individuals over time. Further, this design precludes the ability to correlate the improvement in diet quality with changes in diabetes-related morbidity and mortality. Finally, dietary assessment relies on self-report, which could suffer from recall or reporting biases such as social desirability. If lower SES individuals perceive stigma related to accurately reporting lower dietary quality, which may occur if they know they ‘should’ be eating more healthily but are unable to do so, this would tend to bias our results to the null. This study also has several strengths. It used a high-quality, nationally-representative assessment of diet quality, so results are generalizable to the U.S. population of adults with diabetes. Further, the long study period allows adequate power to detect subtle trends that may be missed with shorter follow-up.

## Conclusions

Adults with both diabetes and lower SES and/or food insecurity experience important disparities in diet quality. These disparities have not improved over time, and lower SES individuals are more than a decade behind their peers in realizing dietary improvements. This represents a significant barrier to optimal diabetes management, and a considerable public health concern. Future work that seeks to improve diet quality for low SES individuals will be an important part of a national strategy to improve diabetes care for vulnerable patients.

## Additional file

Additional file 1: **Table S1**: Health Eating Index-2010 Component Score; the data comes from the Healthy Eating Index 2010. Table lists components of the HEI-2010. **Table S2**: Participants by Diabetes Diagnostic Criteria by NHANES Cycle; NHANES data. Table shows participants by diabetes diagnostic criteria. **Table S3**: Laboratory Values and Medication Use Among Study Participants; NHANES data. Table provides information on the BMI, blood pressure, laboratory values and medication use of participants **Table S4**: Unadjusted Mean Diet Quality by Education, Income and Food Security Status; NHANES data. Table contains information on the mean unadjusted HEI-2010 score. **Table S5**: Mean and Statistical Testing for Differences in HEI-2010 Score by Education, Income and Food Security for those with Self-Reported Diabetes; NHANES data. The table shows differences in diet quality among participants who were diagnosed with diabetes by self-report. **Table S6**: Mean and Statistical Test for Differences in HEI-2010 Score by Education, Income, and Food Security for those with Laboratory Only Diabetes; NHANES data. Table shows differences in diet quality among participants who were diagnosed with diabetes by laboratory criteria only. **Table S7**: Individual HEI Components Score Among Adults with Diabetes NHANES Year 2013–2014; NHANES data. Table shows the individual HEI-2010 components score for adults with diabetes during the 2013–2014 NHANES year. **Figure S1a**: Box and Whisker Plot of Unadjusted Total HEI Score by Education Category among NHANES 2013–2014 Participants; NHANES data. The figure shows the distribution of total HEI-2010 score by education. **Figure S1b**: Box and Whisker Plot of Unadjusted Total HEI Score by Income Category among NHANES 2013–2014 Participants; NHANES data. Figure shows the distribution of total HEI-2010 by income. **Figure S1c**: Box and Whisker Plot of Unadjusted Total HEI Score by Food Security Status among NHANES 2013–2014 Participants; NHANES data. The figure shows the distribution of total HEI-2010 score by food security status. (DOCX 423 kb)

## Data Availability

The dataset was derived from the following public domain resources: https://www.cdc.gov/nchs/nhanes/index.htm; https://www.cdc.gov/nchs/surveys.htm.

## Abbreviations

ACE: Angiotensin converting enzyme
BMI: Body mass index
CVD: Cardiovascular disease
HEI: Health Eating Index
NHANES: National Health and Nutrition Examination Survey
PIR: Poverty to income ratio
