# Supplementary material for: Diet quality trends among adults with diabetes by socioeconomic status in the U.S.: 1999–2014
Source: BMC Endocr Disord. 2019 May 31;19:54. doi: 10.1186/s12902-019-0382-3 (PMC6544994; doi:10.1186/s12902-019-0382-3)
Supplement: Supplementary file 1 — Table S1: Health Eating Index-2010 Component Score; the data comes from the Healthy Eating Index 2010. Table lists components of the HEI-2010. Table S2: Participants by Diabetes Diagnostic Criteria by NHANES Cycle; NHANES data. Table shows participants by diabetes diagnostic criteria. Table S3: Laboratory Values and Medication Use Among Study Participants; NHANES data. Table provides information on the BMI, blood pressure, laboratory values and medication use of participants Table S4: Unadjusted Mean Diet Quality by Education, Income and Food Security Status; NHANES data. Table contains information on the mean unadjusted HEI-2010 score. Table S5: Mean and Statistical Testing for Differences in HEI-2010 Score by Education, Income and Food Security for those with Self-Reported Diabetes; NHANES data. The table shows differences in diet quality among participants who were diagnosed with diabetes by self-report. Table S6: Mean and Statistical Test for Differences in HEI-2010 Score by Education, Income, and Food Security for those with Laboratory Only Diabetes; NHANES data. Table shows differences in diet quality among participants who were diagnosed with diabetes by laboratory criteria only. Table S7: Individual HEI Components Score Among Adults with Diabetes NHANES Year 2013–2014; NHANES data. Table shows the individual HEI-2010 components score for adults with diabetes during the 2013–2014 NHANES year. Figure S1a: Box and Whisker Plot of Unadjusted Total HEI Score by Education Category among NHANES 2013–2014 Participants; NHANES data. The figure shows the distribution of total HEI-2010 score by education. Figure S1b: Box and Whisker Plot of Unadjusted Total HEI Score by Income Category among NHANES 2013–2014 Participants; NHANES data. Figure shows the distribution of total HEI-2010 by income. Figure S1c: Box and Whisker Plot of Unadjusted Total HEI Score by Food Security Status among NHANES 2013–2014 Participants; NHANES data. The figure shows the distribution of total [file 12902_2019_382_MOESM1_ESM.docx]

Supplemental Tables for “Diet Quality Trends Among Adults with Diabetes by Socioeconomic Status in the U.S.: 1999-2014”

| Supplemental Table 1. Healthy Eating Index-2010 Component Score | | | |
| --- | --- | --- | --- |
| Component | Maximum Points | Standard for Maximum | Standard For Minimum Score for Zero |
| HEI-2010 Total Score | 100 | n/a | n/a |
| Total Fruit | 5 | ≥0.8 cup equiv. per 1000kcal | No Fruit |
| Whole Fruit | 5 | ≥0.4 cup equiv per 1000kcal | No Whole Fruit |
| Total Vegetables | 5 | ≥1.1 cup equiv per 1,000 kcal | No vegetables |
| Greens and Beans | 5 | ≥0.2 cup equiv. per 1,000 kcal | No Dark Green Vegetables or Beans and Peas |
| Whole Grains | 10 | ≥1.5oz equiv per 1000 kcal | No Whole Grains |
| Dairy | 10 | ≥1.3 cup equiv. per 1000 kcal | No Dairy |
| Total Protein Foods | 5 | ≥2.5 oz equiv per 1000 kcal | No Protein Foods |
| Seafood and Plant Proteins | 5 | ≥0.8 oz equiv per 1000 kcal | No Seafood or Plant Proteins |
| Fatty Acid | 10 | (PUFA’s+MUFAs)/SFA≥2.5 | (PUFAs+MUFAs)/SFA≤1.2 |
| Moderation |  |  |  |
| Refined Grains | 10 | ≥1.8oz equiv per 1,000 kcal | ≥4.3oz equiv per 1000 kcal |
| Sodium | 10 | ≤1.1 gram per 1000 kcal | ≥2.09 grams per 1000 kcal |
| Empty Calories | 20 | ≤19% of energy | ≥50% of energy |
| This table was adapted from “Update of the Healthy Eating Index: HEI 2010”^14^  PUFA=Polyunsaturated fatty acids  MUFA=Monounsatruated Fatty Acids  SFA=Saturated Fatty Acids | | | |

| Supplemental Table 2. Participants by Diabetes Diagnostic Criteria by NHANES Cycle | | | | | | | | | |
| --- | --- | --- | --- | --- | --- | --- | --- | --- | --- |
| Variable (N) | Overall | 1999-2000 | 2001-2002 | 2003-2004 | 2005-2006 | 2007-2008 | 2009-2010 | 20112012 | 2013-2014 |
|  |  |  |  |  |  |  |  |  |  |
| Self Report | 4400 | 423 | 439 | 477 | 456 | 690 | 679 | 606 | 630 |
| Labs Only | 1311 | 122 | 151 | 122 | 126 | 224 | 229 | 173 | 164 |
| Fasting Glucose Only | 407 | 31 | 51 | 34 | 36 | 77 | 72 | 51 | 55 |
| *”Self Report” defined by those who answered yes to being told s/he has diabetes by a doctor; “Labs Only” defined by adults diagnosed with diabetes who did not self-report but had either a HbA1c ≥6.5 or fasting plasma glucose ≥126. Fasting glucose only indicates those with fasting plasma glucose ≥126 who do not self-report diabetes and did not have an elevated HbA1c. For this analysis we considered those diagnosed with diabetes on the basis of medications to be a separate category (data not shown). | | | | | | | | | |

| Supplemental Table 3. Laboratory Values and Medication Use Among Study Participants | | | | | | | | | | |
| --- | --- | --- | --- | --- | --- | --- | --- | --- | --- | --- |
| Variable (N) | Overall | 1999-2000 | 2001-2002 | 2003-2004 | 2005-2006 | 2007-2008 | 2009-2010 | 2011-2012 | 2013-2014 |  |
|  | Mean/Percent (95%CI) |  |  |  |  |  |  |  |  |  |
| HBA1C (%) (5628) | 7.26 (7.18-7.34) | 7.78 (7.42-8.15) | 7.36 (7.09-7.62) | 7.19 (6.99-7.39) | 7.18 (6.91-7.45) | 7.15 (6.96-7.35) | 7.10 (6.94-7.27) | 7.35 (7.18-7.52) | 7.18 (7.00-7.35) |  |
| BMI (5694) | 32.8 (32.4-33.1) | 31.9 (31.0-32.8) | 32.0 (30.8-33.3) | 31.9 (30.5-33.3) | 32.8 (31.9-33.7) | 33 (32.1-33.9) | 33.3 (32.4-34.3) | 32.9 (31.9-33.9) | 33.5 (32.6-34.4) |  |
| Avg Systolic (mmHg) (5667) | 131.3 (130.4-132.1) | 134.7 (132.6-136.9) | 132.9 (129.5-136.3) | 133.0 (130.2-135.7) | 133.3 (129.5-137.1) | 130.5 (128.7-132.2) | 128.1 (125.4-130.9) | 129.7 (128.1-131.3) | 130.5 (128.1-132.9) |  |
| Avg Diastolic (mmHg) (5667) | 69.4 (68.7-70.1) | 70.6 (67.9-73.4) | 70.2 (67.5-72.8) | 68.9 (65.2-72.6) | 69.5 (67.5-71.5) | 69.7 (68.4-71.0) | 67.1 (65.3-68.9) | 69.8 (68.5-71.2) | 69.8 (67.9-71.7) |  |
| Cholesterol (mg/dL)  (5553) | 192.5 (190.6-194.4) | 208.3 (204.9-211.8) | 204.7 (196.6-212.8) | 204.9 (200.0-209.9) | 193.9 (187.6-200.2) | 188.4 (182.2-194.5) | 185.8 (182.6-188.9) | 185.4 (180.1-190.6) | 182.7 (178.7-186.8) |  |
| HDL (mg/dL) (5552) | 46.9 (46.3-47.5) | 44.0 (42.3-45.7) | 46.3 (44.7-47.9) | 48.5 (46.7-50.2) | 51.0 (48.3-53.8) | 45.6 (44.4-46.8) | 47.1 (45.7-48.5) | 46.0 (44.3-47.7) | 46.6 (44.9-48.4) |  |
| LDL (mg/dL) (2620) | 107.4 (105.5-109.3) | 122.1 (114-129.6) | 114.7 (110.6-118.8) | 112.6 (105.9-119.3) | 109.6 (103.4-115.8) | 103.7 (97.9-109.6) | 105.3 (99.8-110.7) | 102.7 (96.6-108.9) | 103.1 (100.1-106.12) |  |
| Triglyceride (mg/dL) (2899) | 184.5  (175.2-193.9) | 221.4(201.1-241.7) | 204.3 (160.8-247.8) | 219.5 (180.5-258.4) | 182.7 (157.9-207.5) | 175.7  (160.1-191.4) | 167.8  (146.8-188.9) | 178.4 (155.1-201.7) | 160.3 (131.1-189.5) |  |
| Drug Class (5882) |  |  |  |  |  |  |  |  |  |  |
| None | 35.6% (33.8-37.3) | 39.7% (32.6-47.2) | 39.3% (34.3-44.6) | 36.3% (31.3-41.6) | 36.2% (31.2-41.5) | 37.0% (31.5-43.0) | 35.9% (31.2-40.8) | 32.9% (28.4-37.7) | 31.0% (26.5-36.0) |  |
| Metformin | 16.5% (15.2-17.9) | 9.1% (6.6-12.3) | 11.2% (7.4-16.7) | 9.8% (6.5-17.5) | 14.8% (12.2-17.9) | 13.1% (9.0-18.8) | 17.6% (14.5-21.3) | 20.0% (17.5-22.7) | 28.2% (25.2-31.5) |  |
| Sulfonylurea | 8.1% (7.15-9.18) | 18.7% (14.2-24.1) | 14.4% (11.0-18.7) | 10.1% (6.5-15.4) | 8.0% (5.6-11.4) | 7.3% (4.9-10.6) | 5.5% (3.5-8.6) | 5.1% (3.9-6.7) | 2.7% (1.9-4.0) |  |
| >2 Drugs (no insulin) | 22.3% (20.9-24.0) | 15.7% (11.5-21.1) | 22.1% (19.6-24.9) | 27.3% (23.5-31.4) | 23.7% (18.9-29.2) | 25.5% (22.5-28.7) | 24.0% (20.0-28.4) | 20.9% (16.4-26.3) | 19.2% (14.5-25.1) |  |
| Insulin | 17.4% (16.1-18.9) | 16.7% (11.6-23.5) | 12.8% (8.7-18.4) | 16.5% (12.0-22.3) | 17.3% (14.1-21.0) | 17.1% (12.8-22.5) | 17.0% (13.7-20.9) | 21.1% (17.2-25.6) | 18.7% (15.9-22.0) |  |
| ACEI use(5882) | 48.6% (46.7-50.4) | 30.0% (26.1-34.3) | 41.2% (33.9-48.9) | 47.2% (42.1-52.4) | 50.3% (46.0-54.7) | 51.6% (46.0-57.1) | 55.0% (49.1-60.8) | 51.0% (47.5-54.4) | 53.0% (47.1-58.6) |  |
| Statin use (5882)-yes | 48.6% (46.7-50.4) | 30.0% (26.1-34.3) | 41.2% (33.9-48.9) | 47.2% (42.1-52.4) | 50.3% (46.0-54.7) | 51.6% (46.0-57.0) | 55.0% (49.1-60.8) | 51.0% (47.5-54.5) | 52.9% (47.1-58.6) |  |
| HBA1C=Hemoglobin A1c  BMI=Body Mass Index  HDL=High Density Lipoprotein  LDL=Low Density Lipoprotein  ACEI=Angiotensin-converting-enzyme Inhibitor | | | | | | | | | |  |

| Supplemental Table 4. Unadjusted Mean Diet Quality by Education, Income, and Food Security Status | | | | | | | | | |
| --- | --- | --- | --- | --- | --- | --- | --- | --- | --- |
| Variable (N) | Overall | 1999-2000 | 2001-2002 | 2003-2004 | 2005-2006 | 2007-2008 | 2009-2010 | 20112012 | 2013-2014 |
|  | Mean HEI (95%CI) | Mean HEI (95%CI) | Mean HEI (95%CI) | Mean HEI (95%CI) | Mean HEI (95%CI) | Mean HEI (95%CI) | Mean HEI (95%CI) | Mean HEI (95%CI) | Mean HEI (95%CI) |
| Overall (5882) | 50.9 (50.2-51.5) | 49.5 (47.8-51.2) | 49.3 (46.7-51.9) | 48.6 (46.5 -50.6) | 51.3 (49.9 -52.7) | 51.4 (49.7-53.1) | 51.3 (49.9 -52.6) | 52.1 (50.1 -54.1) | 52.0 (50.4 -53.7) |
| Education (5870) |  |  |  |  |  |  |  |  |  |
| <HS% | 49.2 (48.3-50.2) | 48.3 (45.5 -51.1) | 46.5 (42.4 -50.5) | 46.1 (43.6 -48.6) | 53.4 (50.2 -56.6) | 48.8 (47.1 -50.4) | 50.1 (48.3 -51.9) | 51.7 (49.3 -54.0) | 49.1 (46.5 -51.7) |
| HS% | 50.6 (49.5-51.8) | 48.8 (44.3 -53.2) | 51.9 (48.9 -54.8) | 48.4 (44.9 -51.8) | 50.2 (47.9 -52.5) | 51.2 (48.7 -53.8) | 48.4 (46.7 -50.1) | 53.2 (49.6 -56.7) | 51.7 (49.1 -54.4) |
| >HS% | 52.1 (51.1-53.0) | 52.0 (49.1 -55.0) | 50.2 (47.2 -53.1) | 50.2 (47.2 -53.2) | 50.8 (48.9 -52.7) | 53.3 (50.4 -56.2) | 53.2 (51.3 -55.0) | 51.8 (49.0 -54.7) | 53.3 (51.3-55.3) |
| Poverty to Income Ratio (5357) |  |  |  |  |  |  |  |  |  |
| <100% | 48.5 (47.2 -49.8) | 47.9 (43.4 -52.4) | 46.7 (42.6-50.9) | 46.8 (43.5 -50.2) | 49.1 (45.3 -52.9) | 47.6 (44.3 -50.8) | 47.8 (45.5 -50.1) | 50.5 (46.9 -54.2) | 49.5 (46.2 -52.9) |
| 100-200% | 49.8 (48.7 -51.0) | 49.5 (44.3 -54.7) | 48.3 (45.0 -51.7) | 46.8 (43.5 -50.1) | 51.5 (48.2 -54.9) | 49.8 (46.5 -53.2) | 50.8 (48.5 -53.1) | 52.3 (49.9 -54.8) | 48.7 (46.5 -51.0) |
| >200% | 52.0 (51.2 -52.8) | 51.2 (48.7 -53.7) | 50.2 (47.0 -53.5) | 49.9 (46.7 -53.2) | 51.5 (50.0 -53.0) | 52.8 (50.8 -54.8) | 52.0 (50.2 -53.8) | 52.5 (50.0 -54.9) | 53.8 (52.2 -55.4) |
| Adult Food Security 5791) |  |  |  |  |  |  |  |  |  |
| Food Insecure | 48.5 (47.4 -49.5) | 47.3 (42.4 -52.3) | 47.7 (43.8 -51.5) | 46.8 (43.4 -50.3) | 49.5 (45.4 -53.6) | 48.1 (46.0 -50.1) | 48.9 (46.4 -50.5) | 49.8 (47.0 -52.5) | 48.5 (46.4 -50.5) |
| Food Secure | 51.3 (50.6 -52.0) | 49.7 (48.2 -51.3) | 49.4 (46.7 -52.1) | 48.9 (46.7 -51.0) | 51.5 (50.0 -53.1) | 51.8 (50.0 -53.7) | 51.9 (50.5 -53.3) | 52.7 (50.4 -55.0) | 52.7 (50.6 -54.9) |
| *HEI = Healthy Eating Index; HS=High School; Poverty to Income Ratio represents ration of participant’s household income to federal poverty threshold in year of data collection, accounting for household size | | | | | | | | | |

| Supplemental Table 5. Mean and Statistical Testing for Differences in HEI-2010 Score by Education, Income, and Food Security for those with Self-Reported Diabetes | | | | | | |
| --- | --- | --- | --- | --- | --- | --- |
| Raw Overall Mean HEI Score (95% CI) | Adjusted Overall Mean HEI Score (95%CI) |  | | | | |
| 51.7 (51.0 to 52.4) | 52.1 (51.5 to 52.8) |  | | | | |
| Statistical Testing | | | | | | |
| Socioeconomic Indicator | Difference from Reference Group (95% CI) | p-value | Change in HEI-2010 per year (95% CI) | Trend p-value | Interaction Coefficient (95% CI) | Interaction p-value |
| Education |  |  | 0.11 ( -0.04 to 0.25) | 0.14 |  |  |
| <HS | Ref | n/a |  |  | Ref | n/a |
| HS | 3.31 (1.83 to 4.79( | <.001 |  |  | -0.09 (-0.38 to 0.20) | 0.54 |
| >HS | 4.52 (3.16 to 5.88) | <.001 |  |  | -0.01 (-0.29 to 0.27) | 0.94 |
| Poverty to Income Ratio |  |  | 0.13 (-0.02 to 0.28) | 0.09 |  |  |
| <100% | Ref | n/a |  |  | Ref | n/a |
| 100-200% | 0.33 (-1.50 to 2.17) | 0.72 |  |  | -0.12 (-0.51 to 0.27) | 0.56 |
| >200% | 3.81 (2.30 to 5.31) | <.001 |  |  | -0.06 ( -0.38 to 0.27) | 0.73 |
| Food Security Status |  |  | 0.18 (0.03 to 0.32) | 0.02 |  |  |
| Food Insecure | Ref | n/a |  |  | Ref | n/a |
| Food Secure | 2.31 (0.82 to 3.80) | 0.003 |  |  | 0.15 (-0.18 to 0.49) | 0.37 |
| *HEI-2010 = Healthy Eating Index 2010; HS=High School; Poverty to Income Ratio represents a ratio of participant’s household income to federal poverty threshold in year of data collection, accounting for household size. Results are from weighted linear regression model with an interaction term between socioeconomic exposure and NHANES cycle, and adjusted for race/ethnicity, year, age and gender. Models incorporate survey design information for standard errors and use dietary weights for representativeness. P-values are from t-statistics of regression coefficients. NHANES cycle was treated as an ordered categorical variable. | | | | | | |

| Supplemental Table 6. Mean and Statistical Testing for Differences in HEI-2010 Score by Education, Income, and Food Security for those with Laboratory Only Diabetes | | | | | | |
| --- | --- | --- | --- | --- | --- | --- |
| Raw Overall Mean HEI Score (95% CI) | Adjusted Overall Mean HEI Score (95%CI) |  | | | | |
| 48.0 (47.0 to 49.1) | 48.6 (47.5 to 49.8) |  | | | | |
| Statistical Testing | | | | | | |
| Socioeconomic Indicator | Difference from Reference Group (95% CI) | p-value | Change in HEI-2010 per year (95% CI) | Trend p-value | Interaction Coefficient (95% CI) | Interaction p-value |
| Education |  |  | 0.36 (0.11 to 0.60) | 0.005 |  |  |
| <HS | Ref | n/a |  |  | Ref | n/a |
| HS | 0.86 (1.45 to 3.17) | 0.47 |  |  | 0.22 (-0.30 to 0.74) | 0.41 |
| >HS | 2.12 (-0.14 to 4.38) | 0.07 |  |  | -0.26 (-0.76 to 0.75) | 0.33 |
| Poverty to Income Ratio |  |  | 0.36 (0.10 to 0.62) | 0.008 |  |  |
| <100% | Ref | n/a |  |  | Ref | n/a |
| 100-200% | 1.83 (-0.85 to 4.52) | 0.18 |  |  | -0.22 (-0.95 to 0.52) | 0.56 |
| >200% | 3.04 (0.65 to 5.42) | 0.01 |  |  | -0.15 (-0.66 to 0.35) | 0.55 |
| Food Security Status |  |  |  |  |  |  |
| Food Insecure | Ref | n/a | 0.41 | 0.003 | Ref | n/a |
| Food Secure | 1.95 (-0.34 to 4.25) | 0.10 |  |  | 0.13 (-0.46 to -0.73) | 0.66 |
| *HEI-2010 = Healthy Eating Index 2010; HS=High School; Poverty to Income Ratio represents a ratio of participant’s household income to federal poverty threshold in year of data collection, accounting for household size. Results are from weighted linear regression model with an interaction term between socioeconomic exposure and NHANES cycle, and adjusted for race/ethnicity, year, age and gender. Models incorporate survey design information for standard errors and use dietary weights for representativeness. P-values are from t-statistics of regression coefficients. NHANES cycle was treated as an ordered categorical variable. | | | | | | |

| Supplemental Table 7. Individual HEI Components Score Among Adults with Diabetes NHANES Year 2013-2014 | | | | | | | | | | | | |
| --- | --- | --- | --- | --- | --- | --- | --- | --- | --- | --- | --- | --- |
| Characteristic (N) | HEI-2010 Vegetables | HEI-2010 Greens and Beans | HEI-2010 Total Fruit | HEI-2010 Whole Fruit | Whole Grains | Dairy | Total Protein Foods | Seafood and Plant Protein | Fatty Acid Ratio | Sodium | Refined Grains | SOFAAS Calories |
|  | Mean (95% CI) |  |  |  |  |  |  |  |  |  |  |  |
| Overall (836) | 3.1 (2.9 - 3.2) | 1.2 (1.0 -1.5) | 2.0 (1.8 -2.2) | 2.1 (1.8 -2.3) | 2.9 (2.6 -3.3) | 5.0 (4.6 -5.4) | 4.3 (4.1 -4.4) | 2.2 (1.9 -2.5) | 5.2 (4.7 -5.7) | 3.9 (3.6 -4.2) | 6.0 (5.8 -6.3) | 14.2 (13.4 -15.0) |
| Education (835) |  |  |  |  |  |  |  |  |  |  |  |  |
| <HS | 2.8 (2.6 - 3.0) | 1.0 (0.7 -1.4) | 2.0 (1.7 -2.3) | 1.9 (1.6 -2.3) | 2.6 (2.0 -3.2) | 5.4 (4.7 -6.1) | 4.3 (4.1 -4.4) | 1.8 (1.4 -2.1) | 4.6 (4.0 -5.2) | 3.7 (3.4 -4.1) | 5.1 (4.7 -5.6) | 13.8 (12.7 -15.0) |
| HS | 3.0 (2.6 - 3.4) | 1.3 (0.8 -1.8) | 1.8 (1.4 -2.3) | 2.1 (1.6 -2.5) | 2.9 (2.1 -3.7) | 5.0 (4.2 -5.8) | 4.1 (3.8 -4.4) | 2.1 (1.5 -2.7) | 5.6 (4.5 -6.6) | 3.7 (2.8 -4.6) | 6.0 (5.1 -6.8) | 14.2 (12.3 -16.1) |
| >HS | 3.2 (3.0 - 3.4) | 1.3 (1.0 -1.6) | 2.0 (1.7 -2.4) | 2.1 (1.8 -2.5) | 3.1 (2.6 -3.5) | 4.9 (4.4 -5.3) | 4.3 (4.2 -4.5) | 2.4 (2.1 -2.7) | 5.3 (4.7 -5.8) | 4.0 (3.8 -4.4) | 6.4 (6.1 -6.7) | 14.3 (13.6 -14.9) |
| PIR (783) |  |  |  |  |  |  |  |  |  |  |  |  |
| <100% | 2.9 (2.6 - 3.2) | 1.3 (0.8 -1.8) | 1.7 (1.4 -2.0) | 1.7 (1.5 -2.0) | 2.0 (1.5 -2.5) | 5.5 (4.7 -6.2) | 4.3 (4.0 -4.5) | 2.0 (1.6 -2.4) | 5.1 (4.4 -5.9) | 3.5 (3.0 -3.9) | 5.4 (4.7-6.1) | 14.2 (12.5 -15.9) |
| 100-200% | 3.0 (2.7 - 3.3) | 1.1 (0.8 -1.3) | 2.1 (1.7-2.4) | 1.9 (1.6 -2.3) | 2.6 (2.2 -3.1) | 4.6 (3.9 -5.3) | 4.2 (3.9 -4.4) | 1.9 (1.5 -2.2) | 4.8 (4.0 -5.6) | 4.1 (3.5-4.6) | 5.7 (5.2 -6.2) | 12.9 (12.0 -13.8) |
| >200% | 3.2 (3.0 - 3.5) | 1.3 (1.0 -1.6) | 1.9 (1.6 -2.3) | 2.1 (1.8-2.4) | 3.3 (2.7 -3.8) | 5.1 (4.6 -5.5) | 4.3 (4.1 -4.4) | 2.4 (2.0 -2.8) | 5.4 (4.8 -5.9) | 3.9 (3.5 -4.4) | 6.3 (5.8 -6.9) | 14.6 (13.5 -15.8) |
| Food Security (830) |  |  |  |  |  |  |  |  |  |  |  |  |
| Food Insecure | 2.8 (2.4 - 3.1) | 1.2 (0.9 -1.5) | 1.9 (1.6-2.2) | 1.8 (1.3 -2.2) | 2.4 (1.6 -3.2) | 5.4 (4.6 -6.2) | 3.90 (3.7 -4.2) | 1.8 (1.3 -2.3) | 4.6 (3.9 -5.3) | 4.2 (3.7 -4.8) | 5.1 (4.5 -5.8) | 13.3 (12.3 -14.4) |
| Food Secure | 3.1 (3.0 - 3.3) | 1.2 (1.0 -1.5) | 2.0 (1.8 -2.2) | 2.1 (1.9 -2.4) | 3.1 (2.7 -3.4) | 4.9 (4.6 -5.3) | 4.3 (4.2 -4.4) | 2.3 (1.8 -2.7) | 5.3 (4.8 -5.9) | 3.8 (3.5 -4.2) | 6.2 (5.8 -6.5) | 14.3 (13.4 -15.3) |
| *Healthy Eating Index 2010 is comprised of the 12 individual components listed in the table. The range of possible scores on the HEI-2010 is 0-100 with a higher school indicating better dietary quality. Range of score for total fruit, whole fruit, total vegetables, greens and beans, total protein foods and seafood and plant proteins is 0-5 which a higher score indicating higher dietary quality. Range of scores for whole grains, diary, fatty acids, sodium and refined grains is f 0-10 with a higher school indicating higher dietary quality. Range of scores for solid fats, alcoholic beverages & added sugars (SoFAAS) or “empty calories” is 0-20 with a higher score indicating fewer empty calories^15^ | | | | | | | | | | | | |


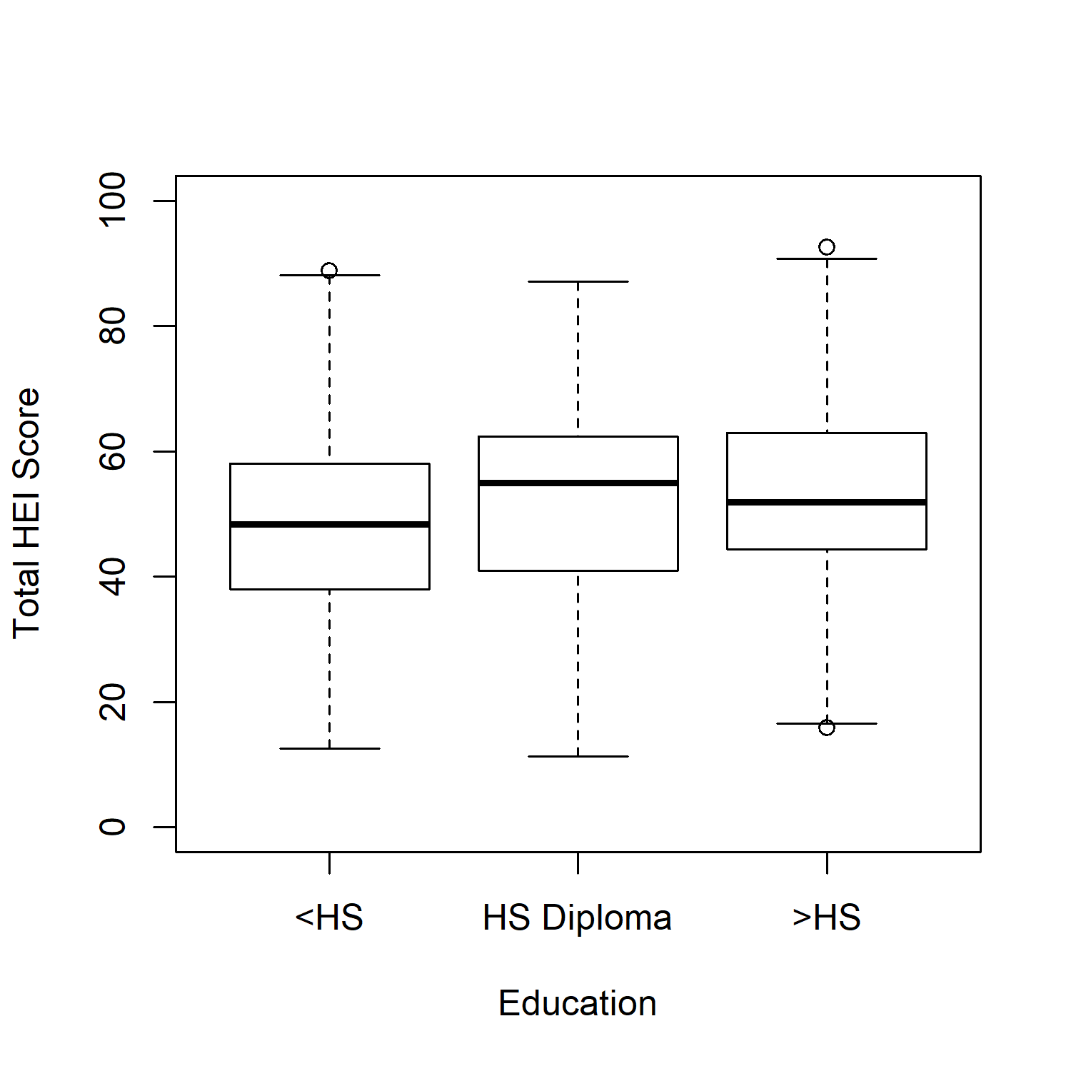
Supplemental Figure 1a: Box and Whisker Plot of Unadjusted Total HEI Score by Education Category among NHANES 2013-2014 Participants

Supplemental Figure 1a Legend: box edges represent quartile 1 and quartile 3, with median drawn as dark line within box. Whiskers extend to extremes of the distribution. HEI = healthy eating Index. HS = high school

Supplemental Figure 1b: Box and Whisker Plot of Total Unadjusted HEI Score by Income Category among NHANES 2013-2014 Participants


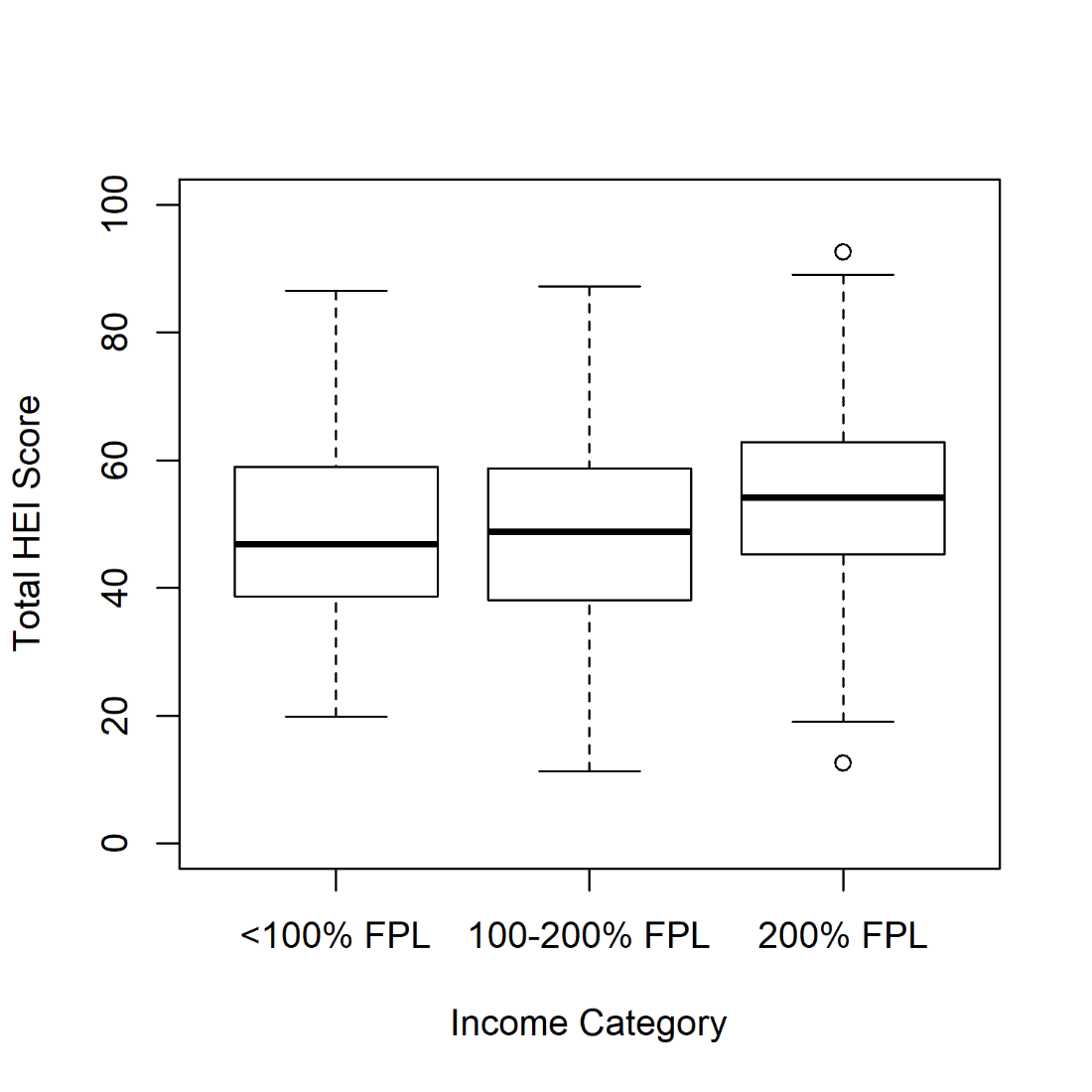


Supplemental Figure 1b Legend: box edges represent quartile 1 and quartile 3, with median drawn as dark line within box. Whiskers extend to extremes of the distribution. HEI = healthy eating Index.

Supplemental Figure 1c: Box and Whisker Plot of Total Unadjusted HEI Score by Income Category among NHANES 2013-2014 Participants


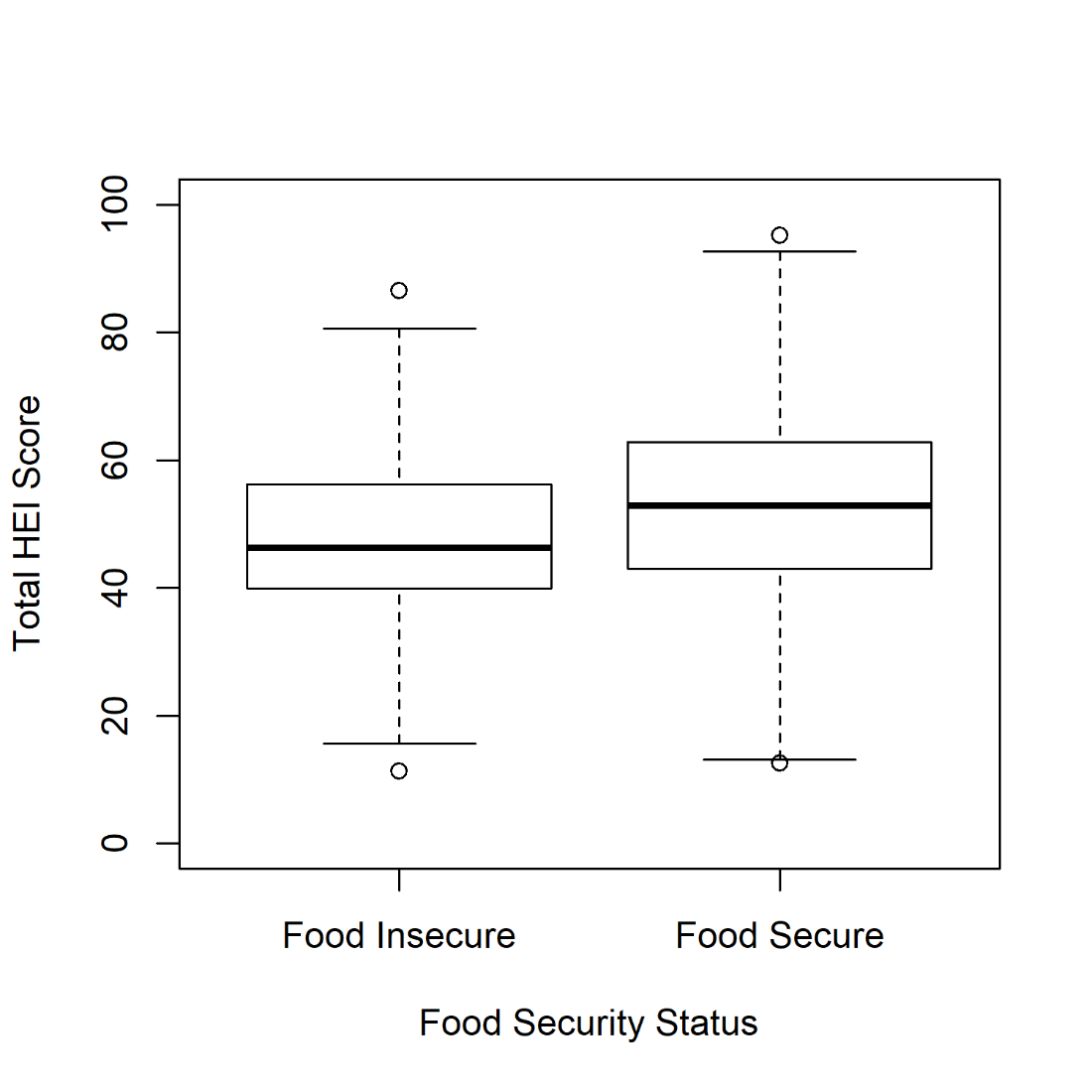


Supplemental Figure 1c Legend: box edges represent quartile 1 and quartile 3, with median drawn as dark line within box. Whiskers extend to extremes of the distribution. HEI = healthy eating Index.
